# Emerging Roles of Mucosal-Associated Invariant T Cells in Rheumatology

**DOI:** 10.3389/fimmu.2022.819992

**Published:** 2022-03-04

**Authors:** Yanmei Li, Jun Du, Wei Wei

**Affiliations:** Department of Rheumatology and Immunology, Tianjin Medical University General Hospital, Tianjin, China

**Keywords:** mucosal-associated invariant T cell, rheumatic disease, systemic lupus erythematosus, rheumatoid arthritis, cytokine

## Abstract

Mucosal-associated invariant T (MAIT) cells are an unconventional T cell subset expressing a semi-invariant TCR and recognize microbial riboflavin metabolites presented by major histocompatibility complex class 1-related molecule (MR1). MAIT cells serve as innate-like T cells bridging innate and adaptive immunity, which have attracted increasing attention in recent years. The involvement of MAIT cells has been described in various infections, autoimmune diseases and malignancies. In this review, we first briefly introduce the biology of MAIT cells, and then summarize their roles in rheumatic diseases including systemic lupus erythematosus, rheumatoid arthritis, primary Sjögren’s syndrome, psoriatic arthritis, systemic sclerosis, vasculitis and dermatomyositis. An increased knowledge of MAIT cells will inform the development of novel biomarkers and therapeutic approaches in rheumatology.

## Introduction

Rheumatic diseases are a group of multisystem, immune-mediated disorders, including rheumatoid arthritis (RA), systemic lupus erythematosus (SLE), primary Sjögren’s syndrome (pSS), psoriatic arthritis (PsA), systemic sclerosis (SSc), large vessel vasculitis and systemic vasculitis. Although the pathogenesis of rheumatic diseases remains unclear, various immune cells, especially T cells, participate in the development of rheumatic diseases (1–5). Mucosal-associated invariant T (MAIT) cells are innate-like T lymphocytes that express a semi-invariant T cell receptor (TCR). MAIT cells are abundant in humans and can respond to pathogens. While many studies have focused on the roles of MAIT cells in infection, it is increasingly clear that they are also involved in autoimmune diseases. In this review, we first summarize the phenotype, distribution, development, activation and function of MAIT cells, and we then describe their roles in rheumatic diseases.

### Phenotype of MAIT Cells

**Figure 1:**
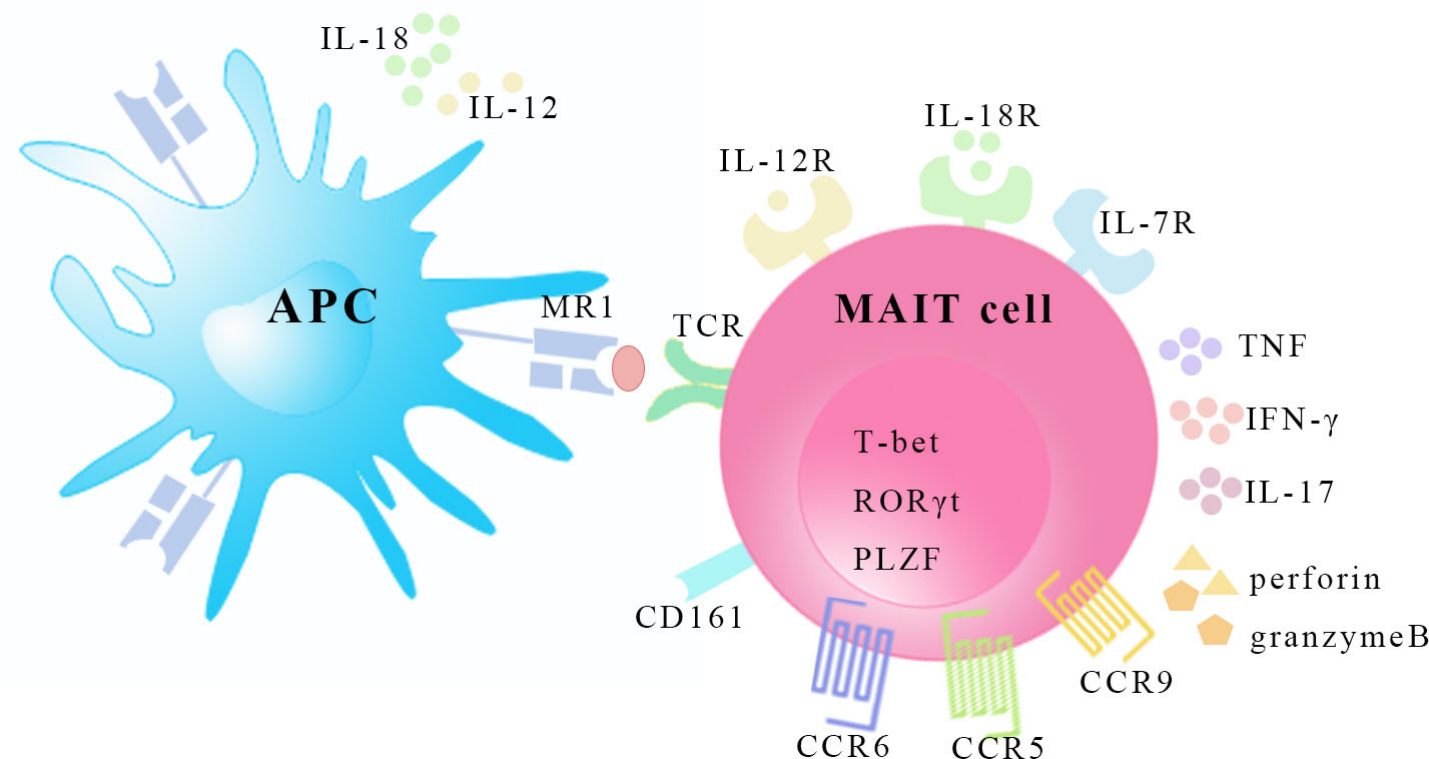
The characteristics of MAIT cells. MAIT cells recognize riboflavin-derived antigens presented by MR1 on surface of antigen presenting cell. MAIT cells express homing receptors CCR5, CCR6 and CCR9, and possess transcription factors T-bet, RORγt, and PLZF. MAIT cells can be activated in a TCR-dependent manner and cytokine-mediated manner by inflammatory cytokines like IL-12, IL-18 and IL-7. In response, MAIT cells secret Th1 and Th17 type cytokines and release granzyme B and perforin. APC, antigen presenting cell. MR1, major histocompatibility complex class 1-related molecule; TCR, T-cell receptor; CCR, CC-chemokine receptor; T-bet, T-Box transcription factor TBX21; RORγt, retinoic-acid-related orphan receptor γt; PLZF, promyelocytic leukemia zinc finger; IL, interleukin; TNF, tumor necrosis factor, IFN-γ, interferon-γ.

Vα7.2-Jα33 (TRAV1-2-TRAJ33) rearrangement was found to be enriched in human double-negative (CD4^-^CD8^-^, DN) α/β T cells and the phylogenetically conserved T cell population was described to express an invariant TCR combined with a limited set of Vβ chains in 1990s (6, 7). In mice, these T cells express the orthologous Vα19-Jα33 (TRAV1-TRAJ33) (7). Since 2003, the new T cell subsets were named mucosal-associated invariant T (MAIT) cells because they are present in mucosal tissues (8). Human MAIT cell subsets are mostly CD8α^+^ (median 79.7%) or DN (median 14.3%) with a minor subset being CD4^+^ cells (median 1.3%) (9), whereas most murine MAIT cells are DN although CD4^+^ and CD8^+^ subsets are detected in lower proportions and with varied frequencies in a tissue-specific manner (10). A recent study has found that human DN and CD8^+^ MAIT cells are functionally distinct subsets, and it is speculated that apoptosis-prone DN MAIT cells may be derived from the main CD8^+^ MAIT cell population (11). MAIT cells recognize metabolites from the bacterial riboflavin (vitamin B2) pathway that are presented by the major histocompatibility complex class 1-related molecule (MR1) (12, 13). MR1 tetramers have been developed for the specific identification of MAIT cells (14). MAIT cells have high surface expression of natural killer (NK) receptors such as C-type lectin CD161, NKG2D and NKP30 (15). The expression of surface markers, including CD45RO, CD95hi, CD27, CD26hi, CD44hi, CD122 and CD62Llo, identify MAIT cells as effector and memory phenotypes (16, 17). MAIT cells also possess high levels of specific homing receptors, such as chemokine CC receptor (CCR)6, CCR5, CCR9, chemokine CXC receptor (CXCR)6 and CXCR3, which are involved in cell migration to peripheral tissues, particularly the liver, intestine and inflammatory tissues (10, 15). However, the expression levels of CXCR2 (CD182), CXCR7, and lymph node homing receptor CCR7 (CD197) are low (18). Moreover, the high surface expression of a series of cytokine receptors such as interleukin (IL)-7, IL-12, IL-18 and IL-23 receptors is of great importance to MAIT cell activation in the absence of TCR stimulation (10, 19). In humans, the T-Box transcription factor TBX21 (T-bet), retinoicacid-related orphan receptor (ROR)γt and promyelocytic leukemia zinc finger (PLZF) transcription factors are coexpressed, suggesting the type1, type 17, and innate-like functionality of MAIT cells (10, 16, 17) (**Figure 1**).

### Distribution and Development of MAIT Cells

MAIT cells are not only enriched in mucosal tissues, but are also abundant in the blood (0.1-9.2%), liver (20-40%) and peripheral lymphoid organs under physiological conditions (8, 9, 20, 21). However, MAIT cells are less frequent in laboratory strains of mice (C57BL/6 and BALB/c) at homeostasis. The proportion is approximately 0.6% of T cells in mouse livers (10). MAIT cells develop in a thymus-dependent manner, similar to humans and mice, and they can be divided into three stages (17). Stage 1 MAIT cells are present only in the thymus and are mostly either double positive (DP) or CD4^+^CD8^-^ thymocytes, and they lack CD161 and CD27 surface markers (CD24^+^CD44^-^ in mice) (17). A small subset of stage 1 MAIT cells express RORγt, which is attributed to vitamin B2 metabolite transferring into the thymus (22). Stage 2 MAIT cells are present at low frequency in the periphery and are mostly CD4^+^CD8^-^ cells that express CD161^-^CD27^+^ (CD24^-^CD44^-^ in mice) with a low expression of T-bet and RORγt (17). MiRNAs may participate in the transition from stage 1 to 2, because in the absence of miR-181a/b-1, thymic MAIT cells show low expressions of ROR γt and T -bet (23). The maturation from stage 2 to stage 3 and acquisition of effector function are PLZF dependent, and commensal bacteria along with IL-18 play an important role in the process (17). Stage 3 MAIT cells acquire functional potential and start to migrate to peripheral organs. At stage 3, MAIT cells expressing CD161^+^CD27^pos-low^ (CD24^-^CD44^+^ in mice) exhibit interferon (IFN)-γ-producing T-bet^+^ (MAIT-1) sublineage or IL-17 A producing RORγt^+^ (MAIT-17) (24). IL-2/IL-15 receptor β chain, inducible costimulatory (ICOS), the transcription factor (TF) Bcl11b and miR-155 are involved in regulating MAIT1 and MAIT17 cells differentiation (25–27). Recently, MAIT2 cells that express PLZF have been defined using single-cell RNA sequencing analysis, which are considered as developmental intermediates of MAIT1 and MAIT17 cells (28). MAIT cells undergo further maturation and expansion after leaving the thymus, and microbes play an important role in regulating the number of MAIT cells in the periphery (29, 30).

### Activation and Function of MAIT Cells

MAIT cells can be activated by two pathways as follows: *via* TCR signalling (MR1 dependent) or cytokine signalling alone (MR1 independent). Riboflavin derivatives presented by MR1 of antigen presenting cells (APCs) activate MAIT cells, while the folic acid product is a competitive inhibitor (31). Because riboflavin synthesis is broadly conserved among bacteria and fungi, MAIT cells respond to a diverse array of microbes, including known commensals (32, 33). Following MR1-mediated activation, MAIT cells rapidly produce a wide range of cytokines, including interferon (IFN)-γ, tumor necrosis factor (TNF), IL-17 and IL-22. These cytokines, either directly (IFN-γ, TNF and IL-17) or indirectly through cell proliferation (IL-22), promote cytolysis of the infected cells, thereby leading to the control of various infections (15, 32, 34, 35). MAIT cells can also be activated by inflammatory cytokines alone, such as IL-7, IL-12, IL-18, IL-15 and IFN-α/β, in a TCR-independent manner (19, 36, 37). MAIT cells are activated with no known antigens, for instance, during viral infections or sterile auto-immunities. Cytokine-dependent activation of MAIT cells leads to secretion of IFN-γ, TNF and granzyme B, which exert cytotoxic functions (36, 37). Transcriptomic analysis of *E. coli*- and TCR-activated MAIT cells has shown distinct transcriptional reprogramming, including altered pathways, transcription factors and effector molecules, which has been validated by proteomics and metabolomics (38). In *E. coli* -activated MAIT cells, the TWIST1 transcription factor has been identified as a key driver, showing high connectivity to cytokine and chemokine expression (38).

## MAIT Cells in Rheumatic Diseases

Because MAIT cells are also involved in a number of inflammatory and autoimmune disorders as well as malignancies, they are attractive targets for therapeutic approaches (39–43). The present paper aims to review the evidence indicating that MAIT cells may contribute to the pathogenesis of rheumatic diseases due to the possible role of MAIT cells in SLE, RA, PsA, ankylosing spondylitis (AS), SSc, pSS, systemic vasculitis, polymyalgia rheumatica and dermatomyositis (**Table 1**).

**Table 1:** MAIT cells in rheumatic diseases.

Disease	Frequency	Phenotype	Function	Reference
SLE	in blood↓ MAIT cells present in kidney with class III and IV disease.	in blood PD-1/CD69/CD25/CD45RA^hi^CD28^-^↑	in blood IFN-γ↓ in animal model, IL-17/IFN-γ↑ in kidney MAIT cells enhance autoantibody production.	(44–46)
RA	in blood↓ synovial fluid↑	in blood CD4^+^↑, CD161↓ CD69↓	in blood and synovial fluid IL-17A↑, IL-23R↑ in animal model, MAIT cells play a pathogenic role.	(44, 47–50)
PsA	in blood↓ synovial fluid↑	in synovial fluid CD8^+^/CD45RO^+^↑	in blood and synovial fluid IL-17A/IL-23R↑ IL-23 promotes IL-17 production by MAIT cells.	(49)
AS	in blood↓ synovial fluid↑	in blood CD69-/↑, in synovial fluid CCR6↑	in blood IL-17↑, IFN-γ↓ in synovial fluid granzyme/IL-17↑, TNF/IFN-γ↓	(47, 51, 52)
pSS	in blood↓ salivary/lacrimal glands↑	in blood CD4^+^↑, CD69/CD154 (CD40L) ↓	in blood TNF/IFN-γ↓, IL-17- in salivary gland IL-17↑ *in vitro* IL-7 and IL-23 induce IL-17 production.	(53, 54)
SSc	in blood↓	ND	ND	(55)
AAV GCA	in blood↓ in GPA and MPA MAIT cells present in arterial wall	in blood CD69↑ in blood CXCR3↓	in blood IFN-γ↑, TNF/IL-17- in blood IFN-γ↑	(56, 57) (58)
PMR	in blood↑ of patients with inactive disease	in blood HLA-DR^+^CD38^+^↑, CD69↑ in remission	ND	(59)
DM	in blood↓	in blood CD25↑, CD45RA^hi^CD28^–^↑	ND	(60)

SLE, Systemic lupus erythematosus; RA, Rheumatoid arthritis; PsA, Psoriatic arthritis; AS, Ankylosing spondylitis; pSS, Primary Sjögren’s syndrome; SSc, Systemic sclerosis; AAV, ANCA-associated vasculitis; GPA, granulomatosis with polyangiitis; MPA, microscopic polyangiitis; GCA, Giant cell arteritis; PMR, Polymyalgia rheumatica; DM, Dermatomyositis; PD-1, programmed death protein 1; IL, interleukin; IFN-γ, interferon-γ; TNF, tumor necrosis factor; CXCR, chemokine CXC receptor; CCR, CC-chemokine receptor; ↑increase; ↓decrease; -comparable; ND, not determined.

### Systemic Lupus Erythematosus

Systemic lupus erythematosus (SLE) is a systemic autoimmune disease characterized by the production of autoantibodies to nuclear antigens. Blood MAIT cell frequency and absolute number are significantly lower in SLE patients than in healthy controls (HCs) (44). Absolute MAIT cell numbers are correlated with age, lymphocyte count, and SLE disease activity index, indicating that MAIT cell levels may reflect disease activity in SLE. The percentage of IFN-γ^+^ MAIT cells is lower in SLE patients than in HCs, and IL-17^+^ or IL-4^+^ MAIT cell levels are similar between SLE patients and HCs (44). IFN-γ production by MAIT cells has been found to be mainly regulated by the nuclear factor of activated T cell (NFAT1) transcription factor, which is intrinsically defective in SLE patients. Moreover, MAIT cell frequency is significantly correlated with NKT cell frequency in SLE patients. In particular, α-GalCer–stimulated NKT cells show poor activation of MAIT cells in SLE patients *in vitro*, suggesting that NKT cell dysfunction influences MAIT cell dysfunction (44). Another study has found that the reduced frequency of MAIT cells in peripheral blood is due to increased cell death, and the expression of activation marker CD69 is correlated with disease activity (45). In addition, monocytes from patients with SLE have an increased capacity to present MR1 antigens and activate MAIT cells. But the antigens that activate MAIT cells in SLE patients remain to be identified. Cytokine analysis has revealed that the plasma levels of IL-6, IL-18 and IFN-α positively correlate with the activated state of MAIT cells in SLE and that MAIT cells are activated by cytokines, including IL-18 and IFN-α, in the absence of exogenous antigens. In the kidneys of SLE patients with class III and IV lupus nephritis, MAIT cells constitute a large proportion of CD3^+^ cells (46). In FcγRIIb^-/-^ *Yaa* mice, an animal model of SLE, more MAIT cells are activated in the kidneys than in the spleen, and the proportions of IL-17- or IFN-γ-expressing MAIT cells are higher in the kidneys than in the spleen. Furthermore, in MR1^-/-^ lupus mice, MAIT cell deficiency results in reduced disease severity, as evidenced by decreased autoantibody production and lower glomerulonephritis scores, and these effects are accompanied by reduced germinal center responses as well as reduced T cell and innate T cell responses (46). Researchers have also demonstrated that MAIT cells contribute to autoantibody production by B cells *in vitro* dependent on the CD40L-CD40 and TCR pathways (46).

### Rheumatoid Arthritis

Rheumatoid arthritis (RA) is a systemic inflammatory disorder and is characterized by chronic, erosive synovitis of diarthrodial joints. Many studies have reported that the MAIT cell percentage is decreased in the peripheral blood of RA patients, and circulating absolute MAIT cell numbers can reflect disease activity in RA (44, 47, 48). The synovial fluid of patients with RA has a higher percentage of MAIT cells than peripheral blood (44, 47). In early, untreated RA patients, the distribution of MAIT cells shifts from CD8^+^ to CD4^+^ MAIT cells, and MAIT cells express lower levels of CD161. Furthermore, CD69 expression of MAIT cells is lower in RA patients compared to HCs, and MAIT cells are hyporesponsive as indicated by minimal upregulation of CD25 and CD69 in response to *E. coli* stimulation (48). Peripheral blood and synovial fluid MAIT cells of RA patients produce more IL-17A compared to osteoarthritis (OA) patients on stimulation (49). In a MAIT cell-deficient MR1^-/-^ mouse model of arthritis, the disease severity of collagen-induced arthritis (CIA) is ameliorated, indicating that MAIT cells play a pathogenic role in RA (50). In addition, MAIT cells augment arthritis mainly by enhancing inflammation in arthritis rather than by a type II collagen-specific response. *In vitro*, it is revealed that IL-1β induces the proliferation of MAIT cells, and IL-23 promotes the production of IL-17 by MAIT cells in the absence of TCR stimulation, indicating that cytokine-mediated MAIT cell activation contributes to the exacerbation of arthritis (50).

### Psoriatic Arthritis

Psoriatic arthritis (PsA) is a chronic inflammatory spondyloarthritis that occurs in combination with psoriasis and has related extraarticular manifestations. The frequency of MAIT cells in peripheral blood is lower in PsA patients than in OA patients, whereas a higher MAIT cell frequency is found in paired synovial fluid, suggesting that MAIT cells are recruited to joints (49). Most MAIT cells in the synovial fluid of PsA patients are of CD8^+^ and the memory (CD45RO^+^) phenotype. Synovial fluid MAIT cells of PsA patients produce significantly more IL-17A than those of RA and OA patients. Moreover, the expression of IL-23R in circulating MAIT cells is higher in PsA and RA patients than in OA patients and IL-23R is functionally active, as evidenced by the profound mitotic effect in the presence of rIL-23 (49). These findings indicate that MAIT cells are likely to be part of the IL-23/IL-17 cytokine network in the pathogenesis of PsA.

### Ankylosing Spondylitis

Ankylosing spondylitis (AS) is a chronic inflammatory rheumatic disease characterized by abnormal ossification and ankylosis affecting the spine and sacroiliac joints. Peripheral blood MAIT cell frequency and number are reduced in AS patients compared with HCs; however, MAIT cell frequency does not correlate with disease activity (47, 51, 52). The results obtained by Gracey et al. show that the level of CD69 of blood MAIT cells in AS patients is equivalent to that in HCs, while Hayashi et al. have found that CD69 expression on circulating MAIT cells correlates with the Ankylosing Spondylitis Disease Activity Score in patients with AS (47, 51). The discrepancy may be due to some factors, such as different disease stages and different treatments. MAIT cells are enriched in synovial fluid with high expression of CCR6. There is a higher percentage of IL-17^+^ and IL-22^+^ MAIT cells and a lower percentage IFN-γ^+^ MAIT cells in the blood of AS patients; in the paired synovial fluid of AS patients, TNF^+^ and IFN-γ^+^ MAIT cells are decreased, and the granzyme B^+^ and IL-17^+^ MAIT cell frequencies are increased (47, 52). Furthermore, IL-17 elevation in circulating MAIT cells of AS patients depends on priming with IL-7 but not IL-23 or MR1-presenting antigens (47).

### Primary Sjögren’s Syndrome

Primary Sjögren’s syndrome (pSS) is a chronic systemic autoimmune disease characterized by lymphocytic infiltration of the exocrine glands leading to glandular dysfunction. pSS patients have a reduced frequency of MAIT cells, and MAIT cells are unable to be identified as a distinct population by CD161 and TCRVα7.2 in half of pSS patients (53). The blood MAIT cells in pSS patients are enriched in the naïve (CD45RA^+^, CCR7^+^) population and show an increase in CD4^+^ subset as well as a decrease in CD8^+^ subset. Compared to MAIT cells in HCs, circulating MAIT cells in pSS patients show lower activation status with lower CD154 and CD69 expression, and they produce less TNF and IFN-γ. There is no difference in the expression of IL-17, Th2 cytokines, granzyme A, granzyme B or perforin between MAIT cells from pSS and control subjects (53). MAIT cells are present in the salivary gland tissue of pSS patients but undetectable in control biopsies (53). However, another study has demonstrated that MAIT cells are IL-17 polarized in the salivary glands of patients with pSS, and that *in vitro* IL-7 and IL-23 stimulation of MAIT cells induces IL-17 over-expression only in pSS patients. In addition, different molecular pathways are explored: IL-7 stimulation induced STAT3/HIF1alpha up-regulation and IL-23 stimulation induced RORc over-expression (54).

### Systemic Sclerosis

Systemic sclerosis (SSc) is an immune-mediated rheumatic disease characterized by immune dysregulation and progressive fibrosis that affects the skin and internal organs. The peripheral blood MAIT cell frequency is decreased in patients with SSc compared to HCs (55). However, the circulating MAIT cell frequency and absolute numbers do not correlate with C reactive protein (CRP), brain natriuretic protein, pulmonary involvement, median skin fibrosis scale, steroid amount or disease duration in patients with SSc.

### ANCA-Associated Vasculitis

Anti-neutrophil cytoplasmic autoantibodies (ANCA)**-**associated (AAV) is an autoimmune disease characterized by inflammation and necrosis of blood vessel walls, which is accompanied by the presence of ANCA antibodies in serum. AAV includes granulomatosis with polyangiitis (GPA), microscopic polyangiitis (MPA) and eosinophilic granulomatosis with polyangiitis (EGPA). The frequency of circulating MAIT cells is lower in patients with AAV compared to HCs, especially in MPA and GPA (56). MAIT cells in peripheral blood from AAV patients in the acute and remission phases exhibit an activated phenotype as judged by their increased expression of CD69 (57). The frequency of circulating MAIT cells that produce IFN-γ is increased in MPA and GPA patients, while the frequency of TNF and IL-17-producing MAIT cells is similar in AAV patients compared to controls (57).

### Giant Cell Arteritis and Polymyalgia Rheumatica

Giant cell arteritis (GCA) is an immune-mediated vasculitis characterized by granulomatous inflammation that affects medium-sized and large arteries. MAIT cells are present in the arterial wall of GCA patients while the percentage of circulating MAIT cells is similar between untreated GCA patients and controls (58). The MAIT cells in GCA patients show decreased expression of CXCR3 and increased expression of IFN-γ. The percentage of circulating IFN-γ^+^ MAIT cells does not decrease after glucocorticoid therapy. MAIT cells from GCA patients proliferate and produce higher amounts of IFN-γ after treatment of IL-12 and IL-18, while the production of granzyme B and TNF is higher after activation of the TCR pathway. Polymyalgia rheumatica (PMR) is an inflammatory rheumatic disease characterized by pain and stiffness in the neck and pelvic girdle, which can occur independently or in association with giant cell arteritis. Among MAIT cells, the percentages of IFN-γ^+^ cells tend to be higher in PMR patients with active disease compared to patients with inactive disease, while the percentages of IL-17^+^ cells are comparable between the active and inactive patient groups. MAIT cells show greater activation in patients with PMR in remission than in controls and active patients as evidenced by a higher frequency of HLA-DR^+^CD38^+^ MAIT cells and CD69^+^ MAIT cells. Moreover, the frequency of HLA-DR^+^CD38^+^ MAIT cells is positively correlated with the PMR activity score and CRP level in patients in remission (59).

### Dermatomyositis

Dermatomyositis (DM) is an inflammatory disorder, characterized by classical cutaneous manifestations and inflammation of skeletal muscle. The frequency of MAIT cells is decreased in active DM patients in comparison with HCs, and the frequency increases after treatment (60). Reduced MAIT cell frequency is associated with increased expression of activation and exhaustion markers such as CD25, CD39 and CTLA4. There is no correlation between MAIT cell frequency and DM clinical scores or biological markers.

## Discussion

MAIT cells were observed to be decreased with an activated phenotype in peripheral blood in patients with rheumatic diseases; whether these changes are a cause or an effect remains to be established. Some studies have found that the frequency or the activation level of MAIT cells is consistent with disease severity (44, 51, 59), indicating that MAIT cells may be potential biomarkers for rheumatic disease. However, longitudinal studies are needed to verify whether the MAIT cell population may recover after treatment. Homing of MAIT cells to inflamed tissues, as evidenced by high expression of chemokine receptors, and activation-induced cell death may contribute to the decrease in circulating MAIT cells. In patients with COVID-19, the frequency of pyroptotic MAIT cells rather than apoptotic MAIT cells is significantly increased, suggesting that activated MAIT cells may exhibit different forms of morbidity (61). Furthermore, a previous study has shown that the CD3^+^ MR1-tetramer^+^ MAIT cell frequency is similar in HCs and patients with RA and that CD161 is reduced in early RA patients, indicating that the gating strategy of CD3^+^CD161^+^Vα7.2^+^ might be partially responsible for the reduction in MAIT cells (48). Another study has also found CD161 is decreased in chronic HIV-1 infection and this Vα7.2^+^CD161^–^ population does not contain MAIT cells (62, 63). Glucocorticoids have long been known to inhibit T cell proliferation and induce apoptosis (64). It has been reported that circulating MAIT cell frequency is associated with oral corticosteroids in asthma patients and MAIT cells are deficient in the airways of steroid-treated chronic obstructive pulmonary disease patients (65, 66). However, no significant correlations are found between circulating MAIT cell levels and use of steroids in SLE and GCA patients (44, 58). This inconsistency could be attributed to different doses of glucocorticoids. More *in vitro* experiments and animal studies are needed to verify the effects of corticosteroids on MAIT cells. MAIT cell activation is observed in some rheumatic diseases and pro-inflammatory cytokines have been identified as potential activator of MAIT cells (45, 57, 59). Is there the possibility that MR1 presents riboflavin derivatives or self-antigens to activate MAIT cells? The use of an inhibitory MR1 ligand to block MAIT cell activation and sterile environments may offer the potential to study the activation mechanism of MAIT cells in rheumatic diseases.

**Figure 2:**
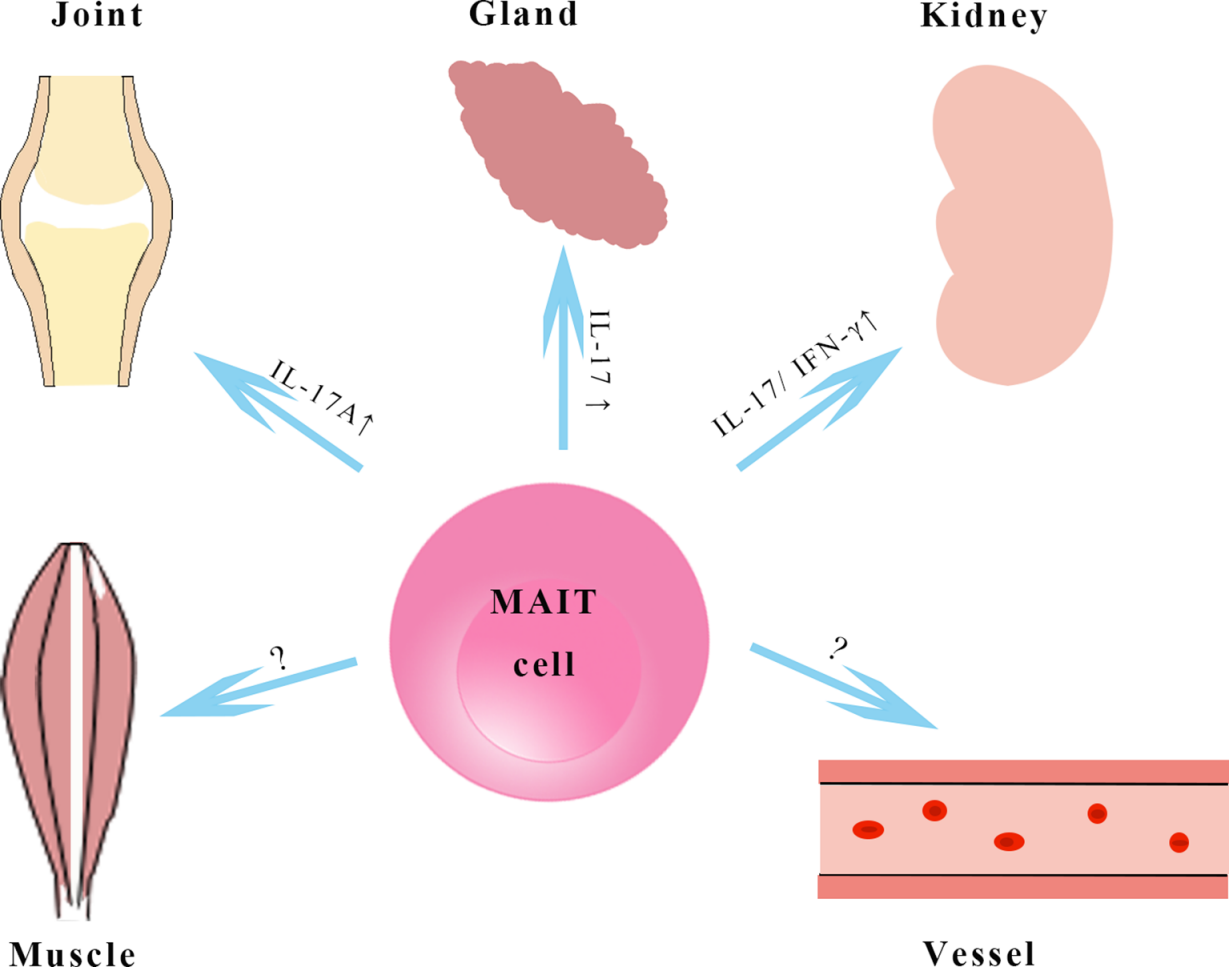
MAIT cells in rheumatological disorders. MAIT cells in kidney, joint, vessels and salivary glands in rheumatic diseases. MAIT cells in tissues produce higher amounts of IL-17 and may exhibit a pathogenic role in animal models with systemic lupus erythematosus and rheumatoid arthritis, however, the functions of MAIT cells in vessels and muscles remain to be elucidated. IL-17, interleukin-17; IFN-γ, interferon-γ.

In most rheumatic diseases, IFN-γ and TNF produced by circulating MAIT cells decrease, while IL-17 produced by MAIT cells increases in blood and tissues (**Figure 2**), suggesting that MAIT cell proinflammatory IL-17 is biased in rheumatology similar to that in obesity and chronic liver disease (67–69). IL-17 plays different pathological roles in some rheumatic diseases, like the promotion and perpetuation of inflammation and the damage to the affected tissues (70, 71). Several therapeutic strategies targeting IL-17 have been approved for the treatment of PsA and AS. MAIT cells, as a source of IL-17, emerge in the mechanism of rheumatology and might be a promising therapeutic option. It remains to be determined whether MAIT cells are “friends or foes” in autoimmune responses. MAIT cells appeared with pathogenic roles in SLE and RA animal models; however, a protective role of MAIT cells in an animal model of multiple sclerosis has been observed (46, 50, 72). In type 1 diabetes, MAIT cells show dual roles in maintaining gut barrier integrity by secreting IL-17A and IL-22 but promote β-cell death in the pancreas (73). The effects of MAIT cells seems to be dependent on disease stages and their tissue localization, and MAIT cells may have other functions we do not yet know about due to the lack of known antigens of MAIT cells. More research is needed to further study the molecular mechanism of MAIT cells in rheumatic diseases, especially the MAIT cells recruited to affected organs. The cross-talk of MAIT cells and several immune cell subsets by secretion of cytokines and chemokines has been demonstrated (74). Our team has found MAIT cells induce anti-inflammatory macrophage polarization by producing regulatory cytokines in nonalcoholic fatty liver disease (75). MAIT cells increase plasmablasts and promote Ig production *in vitro*, and they are involved in autoantibody production by B cells in SLE (46, 76). We speculate that these MAIT-B cell interactions may occur in B cell/autoantibody-driven disorders, such as pSS and AAV. Further studies to understand how MAIT cells encounter autoreactive B cells are needed.

There is increasing evidence that microbiomes and metabolites play a vital role in the immunopathogenesis of rheumatic diseases (77, 78). The reduced richness of microbial species and a reduced ratio of *Firmicutes/Bacteroides* in the gut are common features of rheumatic disease (79). As described above, microbiota-derived metabolites affect MAIT cell selection, expansion and function. In alcoholic liver disease, intestinal bacteria and metabolites drive selective MAIT cell depletion and dysfunction, whereas MAIT cells induce gut microbiota alterations in obesity model (80, 81). Thus, there are a few questions to be answered. It remains unknown whether the intestinal flora and metabolites change first to cause alternation in the functions of MAIT cells, thereby contributing to the diseases. It is important to understand how microbiota affect the phenotype of MAIT cells, and how microbiota-metabolite-MAIT cell interactions shape adaptive immunity. More in-depth investigations are required to study the correlations among dysbiosis, MAIT cell responses and clinical symptoms. The relative abundance of microbiota and metabolites in diseases can be determined by metagenomic and metabolomics analysis. And it would be interesting to investigate whether fecal microbiota transplantation from rheumatic patients in mouse models results in MAIT cell activation and disease exacerbation.

In this review, we have summarized current evidence supporting the emerging roles for MAIT cells in rheumatology. It is imperative that we continue to elucidate the activation mechanisms and functions of MAIT cells to better understand pathogenesis of rhematic diseases. MAIT cell deficiency (MR1^-/-^) and transgenic mice models, as wells as acetyl-6-formylpterin, a potent blocking MAIT cell molecule (82), are the new tools to be used for further investigations. The analysis of the crosstalk between MAIT cells and other immune cells and the interactions between MAIT cells and microbiota will identify therapeutic targets and approaches for clinical intervention in the future.

## Author Contributions

YL and JD wrote the manuscript. WW provided the feedback. All authors contributed to the article and approved the submitted version.

## Funding

This work was supported by the National Natural Science Foundation of China (grant 81900523).

## Conflict of Interest

The authors declare that the research was conducted in the absence of any commercial or financial relationships that could be construed as a potential conflict of interest.

## Publisher’s Note

All claims expressed in this article are solely those of the authors and do not necessarily represent those of their affiliated organizations, or those of the publisher, the editors and the reviewers. Any product that may be evaluated in this article, or claim that may be made by its manufacturer, is not guaranteed or endorsed by the publisher.
